# Supplementary material for: Anti-Inflammatory and Analgesic Effects of TRPV1 Polypeptide Modulator APHC3 in Models of Osteo- and Rheumatoid Arthritis
Source: Mar Drugs. 2021 Jan 17;19(1):39. doi: 10.3390/md19010039 (PMC7830295; doi:10.3390/md19010039)
Supplement: Supplementary file 1 [file marinedrugs-19-00039-s001.pdf]

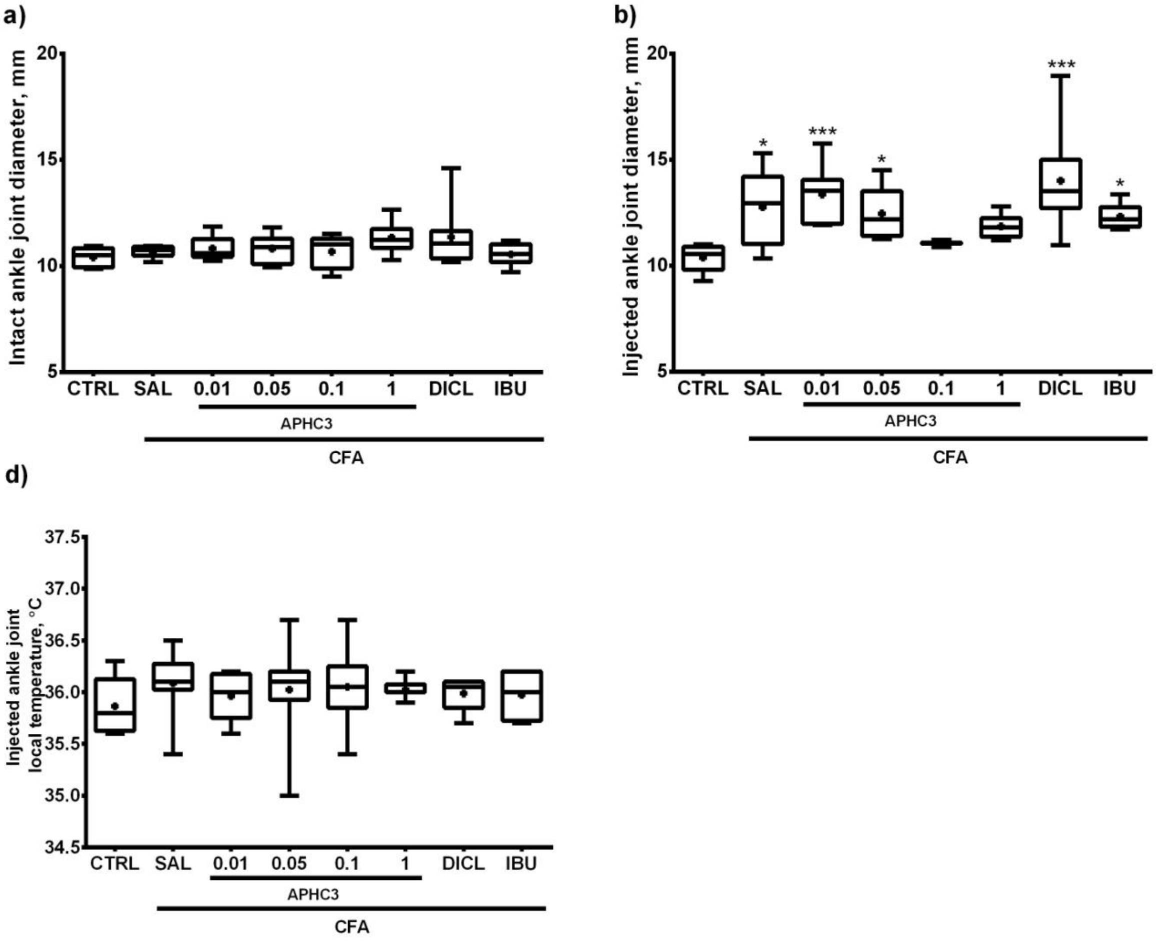

Figure S1 Assessment of inflammation in the ankle joint on the day 3 after intra-articular administration of CFA (40μl) and anti-inflammatory effects of APHC3 (0.01, 0.05, 0.1 and 1 mg/kg s.c.), diclofenac (20 mg/kg i.m.) and ibuprofen (40 mg/kg p.o.). (a) diameters of the contralateral intact joints; (b) absolute and (c) local temperature of CFA-injected joint. Abbreviation CTRL designates control group, SAL - sterile saline, DICL - diclofenac, and IBU - ibuprofen. Results are presented as median and mean is shown as a cross (+), interquartile range, and minimum and maximum (*n* = 8 for each group). Statistical analysis was performed using the Kruskal-Wallis criterion followed by Dunn's multiple comparisons test. * - $p<0.05$ vs CTRL, ** - $p<0.01$ vs CTRL, *** - $p<0.001$ vs CTRL.

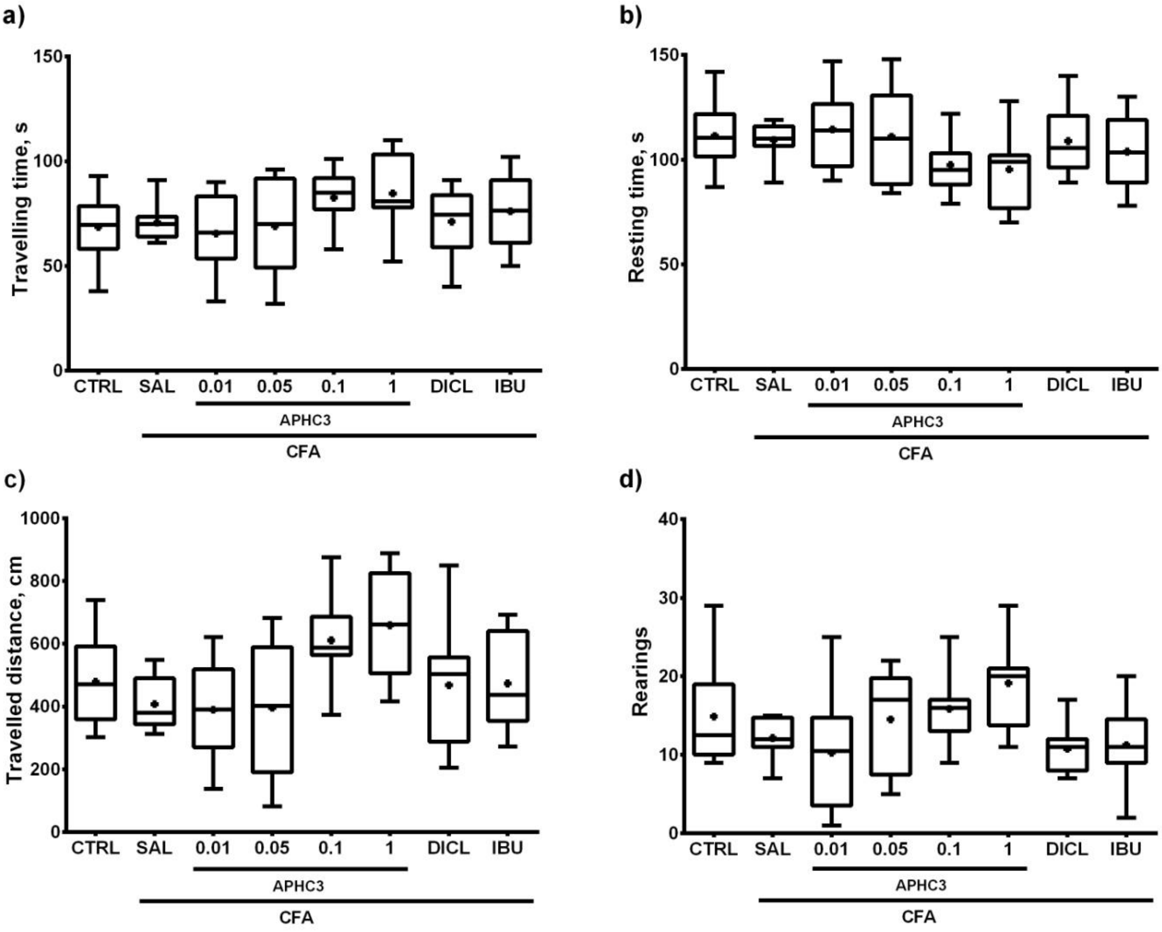

Figure S2. Locomotor activity in the open field on day 3 after intra-articular administration of CFA (40µl). (a) travelling time, (b) resting time, (c) travelled distance and (d) rearing count were not changed after administration of APHC3 (0.01, 0.05, 0.1 and 1 mg/kg s.c.), diclofenac (DICL, 20mg/kg i.m.) and ibuprofen (IBU, 40 mg/kg p.o.). Abbreviation CTRL designates Control group, SAL - sterile saline. Results are presented as median and mean is shown as a cross (+), interquartile range, and minimum and maximum (n = 8 for each group). Statistical analysis was performed using the Kruskal-Wallis criterion followed by Dunn's multiple comparisons test.

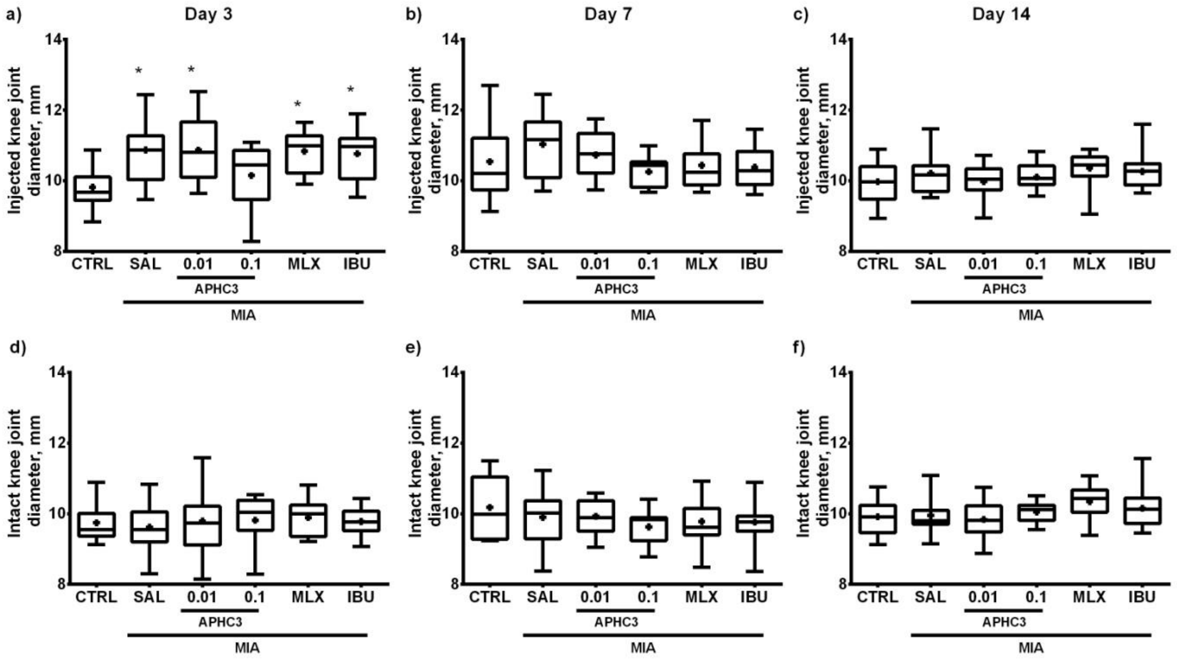

Figure S3. Absolute diameters of the knee joints in the MIA-induced OA model. Diameters (in mm) of the injected (a-c) and intact (d-f) joints were measured on days 3 (a, d), 7 (b, e), and 14 (c, f) after intra-articular MIA injection into the right knee joint (3 mg MIA in 50 µl of sterile saline).. APHC3 (0.01 and 0.1 mg/kg s.c.), meloxicam (0.5 mg/kg i.m.) and ibuprofen (40 mg/kg p.o.) were administered daily on the days 3-14. Abbreviation CTRL designates control group, SAL - sterile saline, MLX - meloxicam, and IBU - ibuprofen. Results are presented as median and mean is shown as a cross (+), interquartile range, and minimum and maximum (*n* = 9-12 for each group). Statistical analysis was performed using the Kruskal-Wallis criterion followed by Dunn's multiple comparisons test. * - $p<0.05$ vs CTRL, ** - $p<0.01$ vs CTRL, *** - $p<0.001$ vs CTRL, $^{\#}$ - $p<0.05$ vs SAL, $^{\#\#}$ - $p<0.01$ vs SAL, $^{\#\#\#}$ - $p<0.001$ vs SAL.

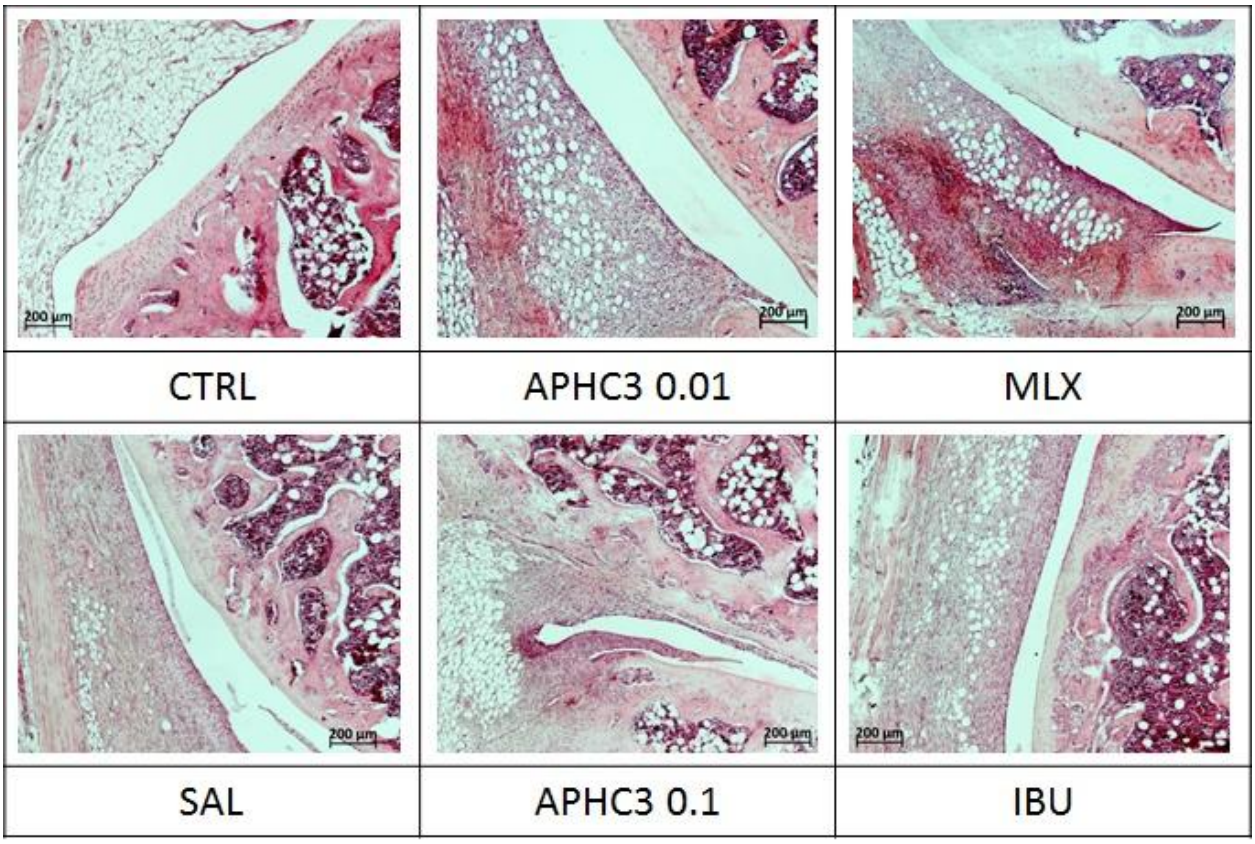

Figure S4. Representative images of the injected knee joint synovia in the MIA-induced OA model. Inflammatory infiltration (InIn) and synovial hyperplasia (SH) were assessed on day 8 after intra-articular MIA injection into the right knee joint (3 mg MIA in 50 µl of sterile saline). Each sign was graded scores on a scale of 0 to 5, where 0 represents a normal tissue and 5 represents severe tissue degeneration. CTRL (control group): InIn 0, SH 0; SAL (sterile saline-treated group): InIn 4, SH 3; APHC3 0.01mg/kg: InIn 3, SH 3; APHC3 0.1mg/kg: InIn 3, SH 2; MLX (meloxicam-treated group): InIn 3, SH 2; IBU (ibuprofen-treated group): InIn 3, SH 2.

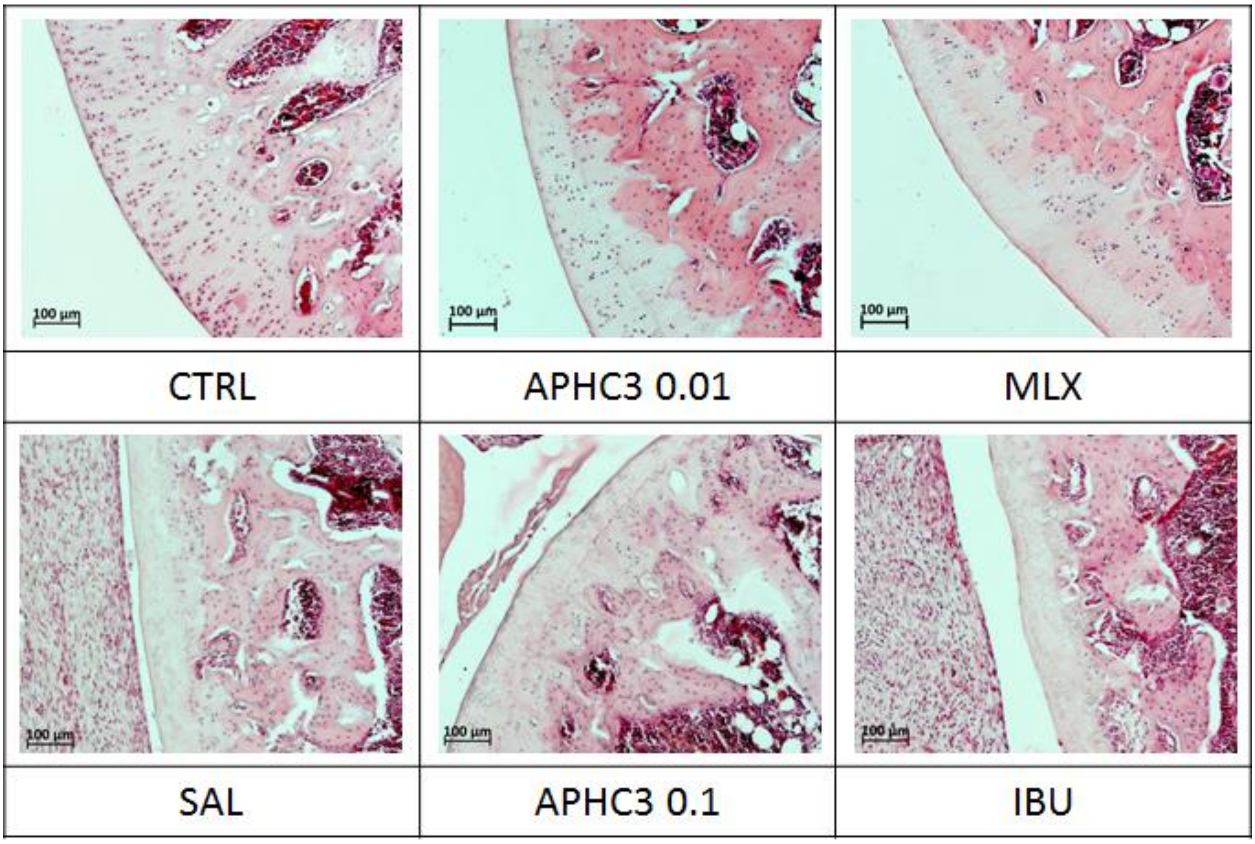

Figure S5 Representative images of the distal femoral cartilage of the injected knee joint in the MIA-induced OA model. Cartilage (CD) and bone destruction (BD) were assessed on day 8 after intra-articular MIA injection into the right knee joint (3 mg MIA in 50 µl of sterile saline). Each sign was graded scores on a scale of 0 to 5, where 0 represents a normal tissue and 5 represents severe tissue degeneration. CTRL (control group): CD 0, BD 0; SAL (sterile saline-treated group): CD 4, BD 1; APHC3 0.01mg/kg: CD 3, BD 0; APHC3 0.1mg/kg: CD 3, BD 0; MLX (meloxicam-treated group): CD 3, BD 0; IBU (ibuprofen-treated group): CD 4, BD 1.

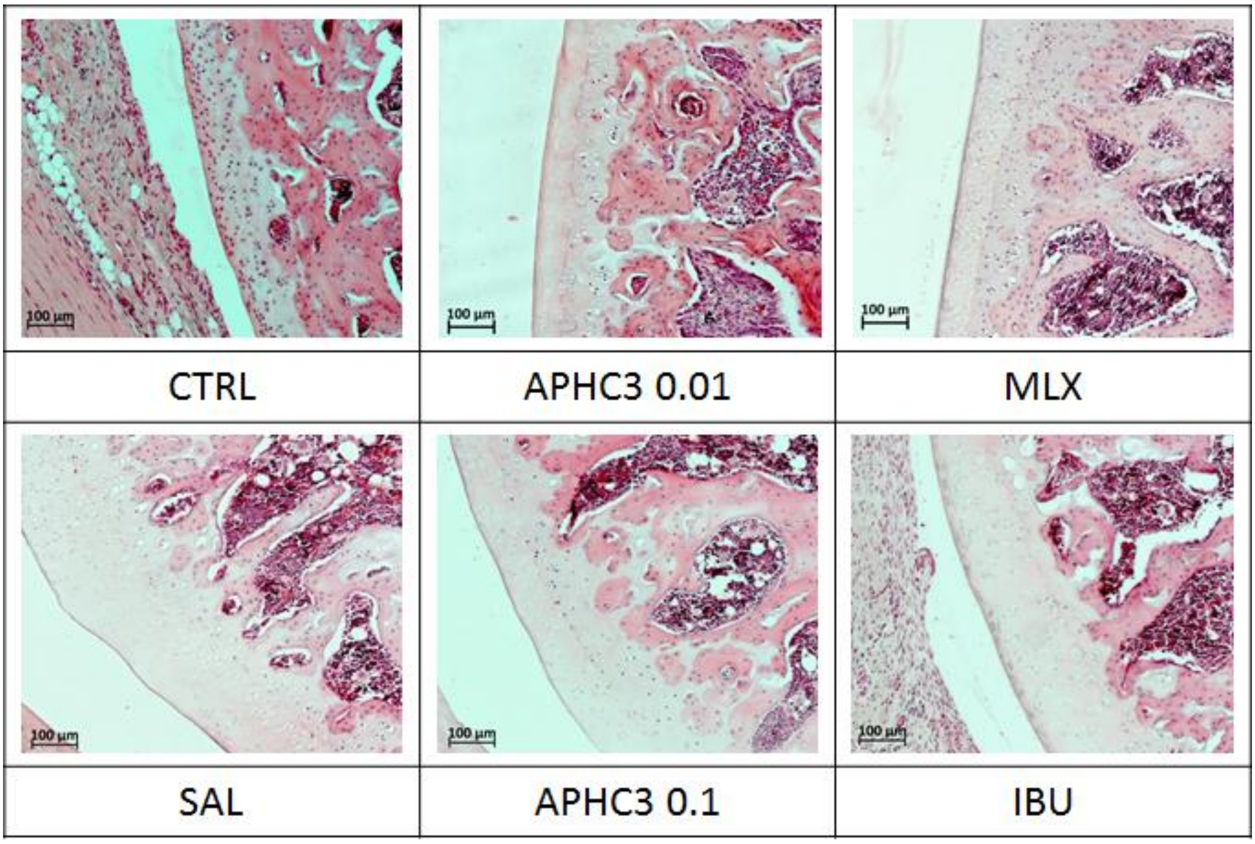

Figure S6 Representative images of the distal femoral cartilage of the injected knee joint in the MIA-induced OA model. Cartilage (CD) and bone destruction (BD) were assessed on day 15 after intra-articular MIA injection into the right knee joint (3 mg MIA in 50 µl of sterile saline). Each sign was graded scores on a scale of 0 to 5, where 0 represents a normal tissue and 5 represents severe tissue degeneration. CTRL (control group): CD 0, BD 0; SAL (sterile saline-treated group): CD 4, BD 0; APHC3 0.01mg/kg: CD 3, BD 0; APHC3 0.1mg/kg: CD 3, BD 0; MLX (meloxicam-treated group): CD 3, BD 0; IBU (ibuprofen-treated group): CD 4, BD 0.

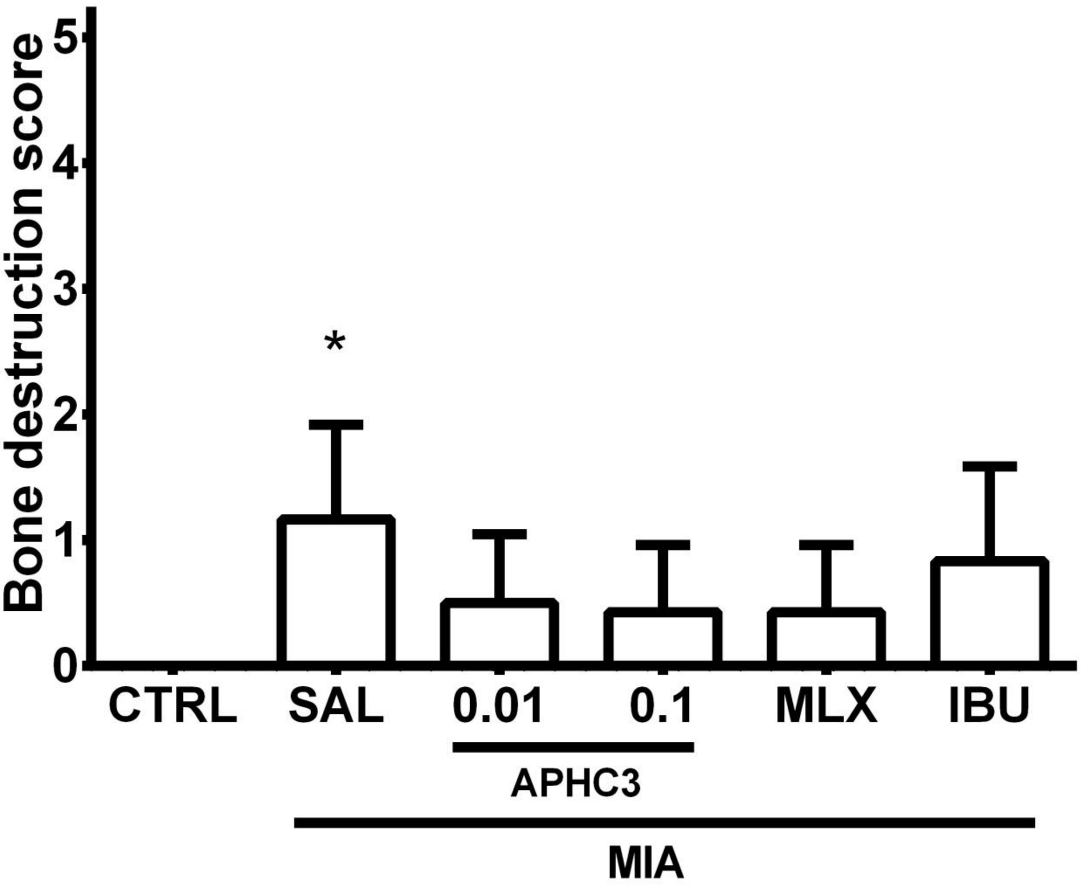

Figure S7. Histological analysis of bone destruction of the injected knee joint in the MIA-induced OA model. Destructive changes were assessed on 15 after intra-articular MIA injection into the right knee joint (3 mg MIA in 50 μl of sterile saline). APHC3 (0.01 and 0.1 mg/kg s.c.), meloxicam (MLX, 0.5 mg/kg i.m.) and ibuprofen (IBU, 40 mg/kg p.o.) were administered daily on the days 3-14. Control and saline-administered groups are denoted as CTRL and SAL, respectively. Results are presented as mean and SD (*n* = 6-7). Statistical analysis was performed using the Kruskal-Wallis criterion followed by Dunn's multiple comparisons test. * - p<0.05 vs CTRL, ** - p<0.01 vs CTRL, *** - p<0.001 vs CTRL, $^{\#}$ - p<0.05 vs SAL, $^{\#\#}$ - p<0.01 vs SAL, $^{\#\#\#}$ - p<0.001 vs SAL.
